# A Case of Large Rice Body Containing Cyst With an Irreparable Rotator Cuff Tear and Hill-Sachs Lesion Treated With Reverse Shoulder Arthroplasty

**DOI:** 10.7759/cureus.101881

**Published:** 2026-01-19

**Authors:** Masashi Koide, Satoshi Tateda, Taishi Murakami, Mika Abe

**Affiliations:** 1 Orthopedic Surgery, Japanese Red Cross Ishinomaki Hospital, Ishinomaki, JPN

**Keywords:** rheumatoid arthritis, rice body, rotator cuff tear, shoulder arthroplasty, shoulder instability

## Abstract

Subacromial-subdeltoid bursitis with rice bodies, associated with shoulder instability, is uncommon. The pathogenesis of rice body formation remains unclear; however, it is considered a nonspecific response to chronic inflammation. We present the case of a 79-year-old woman with subacromial-subdeltoid bursitis of the right shoulder, numerous rice bodies, and rheumatoid arthritis (RA). She had a history of right shoulder dislocation, an irreparable rotator cuff tear, and a large Hill-Sachs lesion. She underwent reverse shoulder arthroplasty (RSA) with rice body removal and showed symptom improvement. RSA is a definitive treatment that can address three key issues: rice bodies caused by RA, rotator cuff deficiency, and instability from humeral bone loss.

## Introduction

Rice bodies are small, smooth, fibrin-rich nodules that arise in response to chronic inflammation of synovial tissue or tendon sheaths and are considered a nonspecific inflammatory phenomenon [1]. Although most frequently associated with rheumatoid arthritis (RA), rice bodies have also been reported in association with traumatic conditions, juvenile idiopathic arthritis, seronegative inflammatory arthropathies, infections, graft reactions, and osteoarthritis [2-4]. Rice body formation has been reported in various joints, with the shoulder representing one of the commonly involved sites, particularly in the setting of rheumatoid arthritis [4,5]. Only a limited number of reports have described subacromial-subdeltoid bursitis with rice bodies as the initial manifestation of RA [6,7].

Rotator cuff tears often coexist with rice body-associated subacromial-subdeltoid bursitis in patients with RA [6]. However, previously reported treatments have mainly consisted of open or arthroscopic bursectomy or corticosteroid injection, without addressing rotator cuff dysfunction [8,9].

Hill-Sachs lesions are frequently encountered in shoulders with anterior instability and represent compression defects of the humeral head resulting from impact against the anterior glenoid rim. The reported incidence of Hill-Sachs lesions ranges from 65%-67% after a first dislocation to 84%-93% following recurrent instability events [10]. The concept of the “glenoid track,” introduced by Itoi, describes the contact zone between the posterior humeral head and the glenoid during shoulder motion [10]. Lesions extending beyond this track (“off-track” lesions) remain at risk of engagement and recurrent dislocation [11].

Herein, we describe an unusual case of extensive rice body formation in the subacromial-subdeltoid bursa combined with shoulder instability secondary to an irreparable rotator cuff tear and a large Hill-Sachs lesion. Reverse shoulder arthroplasty (RSA) was selected to simultaneously manage RA-related rice bodies, rotator cuff insufficiency, and instability caused by humeral bone loss. To the best of our knowledge, this is the first reported case in which RSA was used for the treatment of RA-associated rice body formation.

## Case presentation

**Figure 1:**
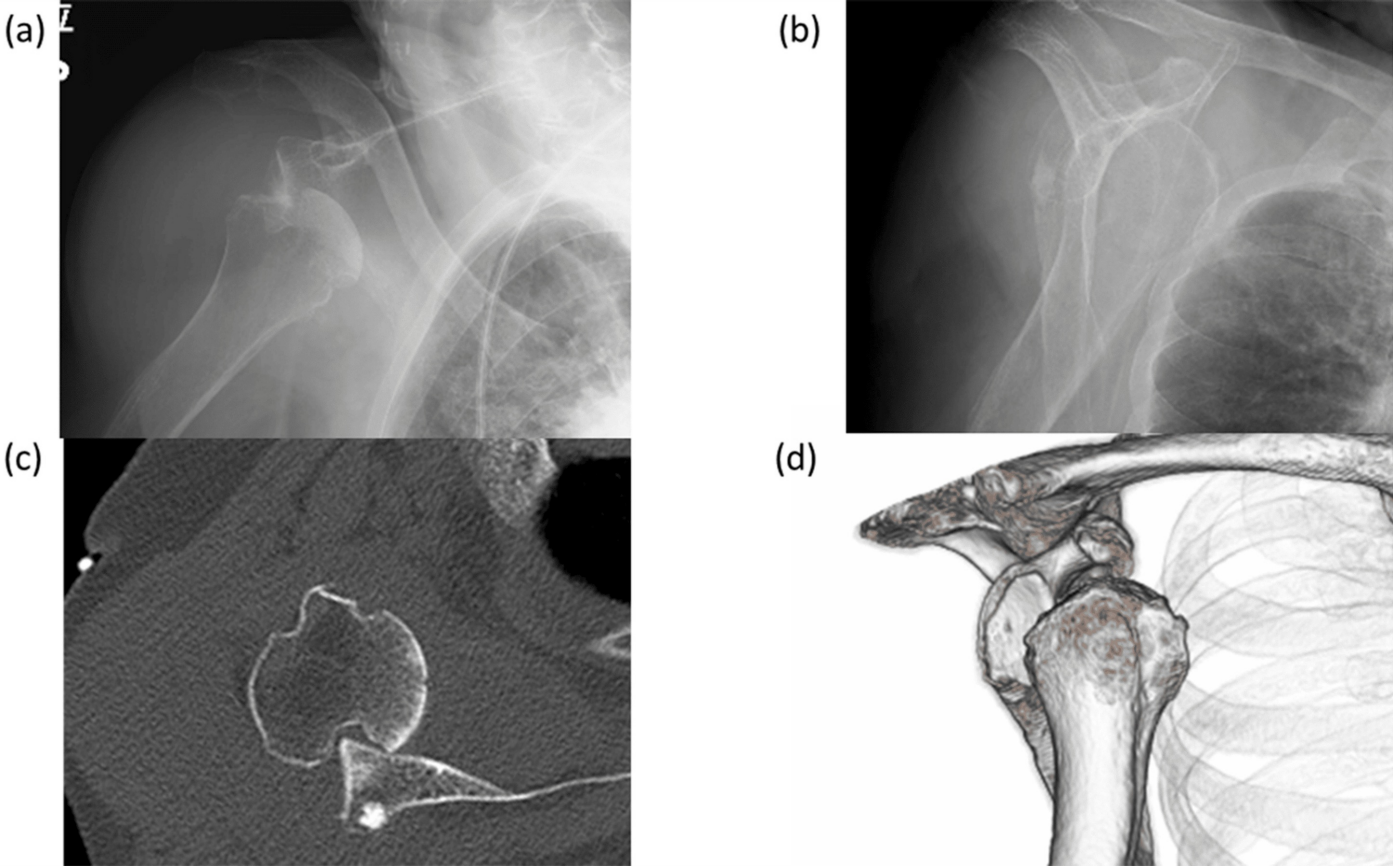
Plain radiographic images and CT on emergency department (a) An anteroposterior radiograph showing shoulder dislocation. (b) A scapular Y view showing anterior shoulder dislocation. (c) CT showing anterior shoulder dislocation and an engaged Hill-Sachs lesion. (d) 3D reconstruction of the CT images. CT: computed tomography

**Figure 2:**
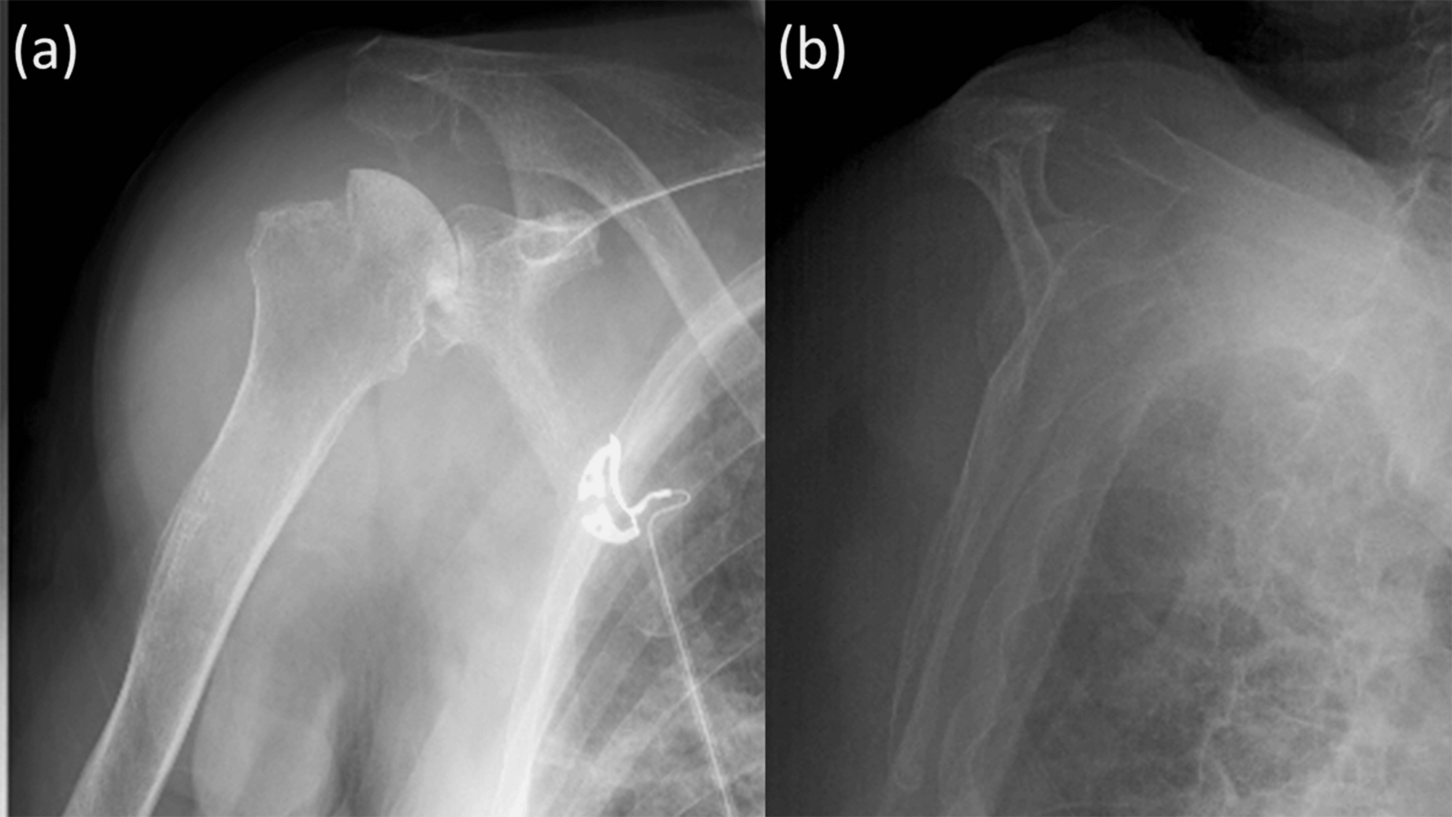
Plain radiographic images after the reduction of shoulder dislocation (a) An anteroposterior radiograph showing a significant Hill-Sachs lesion. (b) A scapular Y view showing a reduced position of the humeral head.

A 79-year-old woman with a known history of RA was brought to the emergency department after losing consciousness due to near drowning in a bathtub. Following cardiopulmonary resuscitation, she regained consciousness. Whole-body computed tomography (CT) revealed anterior dislocation of the right shoulder, accompanied by soft tissue swelling and pneumonia (Figure 1). The shoulder dislocation was manually reduced and immobilized using a sling (Figure 2). She was subsequently admitted for treatment of aspiration pneumonia with intravenous antibiotics. After three weeks of antibiotic therapy, the patient continued to complain of persistent pain and swelling of the right shoulder and was referred to the orthopedic department. She reported progressive shoulder pain and swelling for approximately six months, with gradual enlargement of the swelling. Medical therapy for RA had been discontinued several years earlier because of a gastric ulcer.

**Figure 3:**
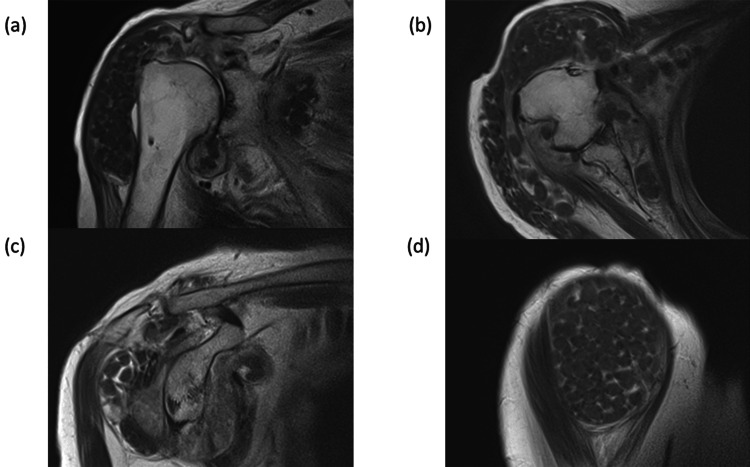
Preoperative MRI (a) Coronal view showing the subacromial-subdeltoid bursa, including numerous rice bodies and massive supraspinatus tendon tears. (b) Axial view showing the subdeltoid bursa, including innumerable rice bodies, large Hill-Sachs lesions, and thin subscapularis/infraspinatus tendon. (c) Sagittal view at the level of the glenoid showing the subacromial-subdeltoid bursa, including innumerable rice bodies and a retracted atrophic rotator cuff muscle. (d) Sagittal view at the level of the deltoid muscle showing the subdeltoid bursa, including numerous rice bodies. MRI: magnetic resonance imaging

On physical examination, localized swelling, tenderness, and limited shoulder motion were observed. Laboratory studies showed a mildly elevated C-reactive protein level of 1.42 mg/L, while the white blood cell count was within the normal range (6.9 × 10⁹/L). Rheumatoid factor (29.6 IU/mL) and anti-cyclic citrullinated peptide antibody levels (125 U/mL) were both elevated. Plain radiographs demonstrated a Hill-Sachs lesion, concentric joint space narrowing, and subchondral sclerosis of the humeral head without a spur. CT imaging confirmed marked soft tissue swelling and a large Hill-Sachs defect (Figure 1). Magnetic resonance imaging (MRI) revealed marked distension of the subacromial-subdeltoid and subcoracoid bursae, containing numerous nodules that were isointense to muscle on T1-weighted sequences, consistent with rice bodies (Figure 3). A massive full-thickness rotator cuff tear allowed communication between the glenohumeral joint and the bursal space. Multifocal bone erosion was evident in the humeral head, glenoid, undersurface of the acromion, and distal clavicle.

**Figure 4:**
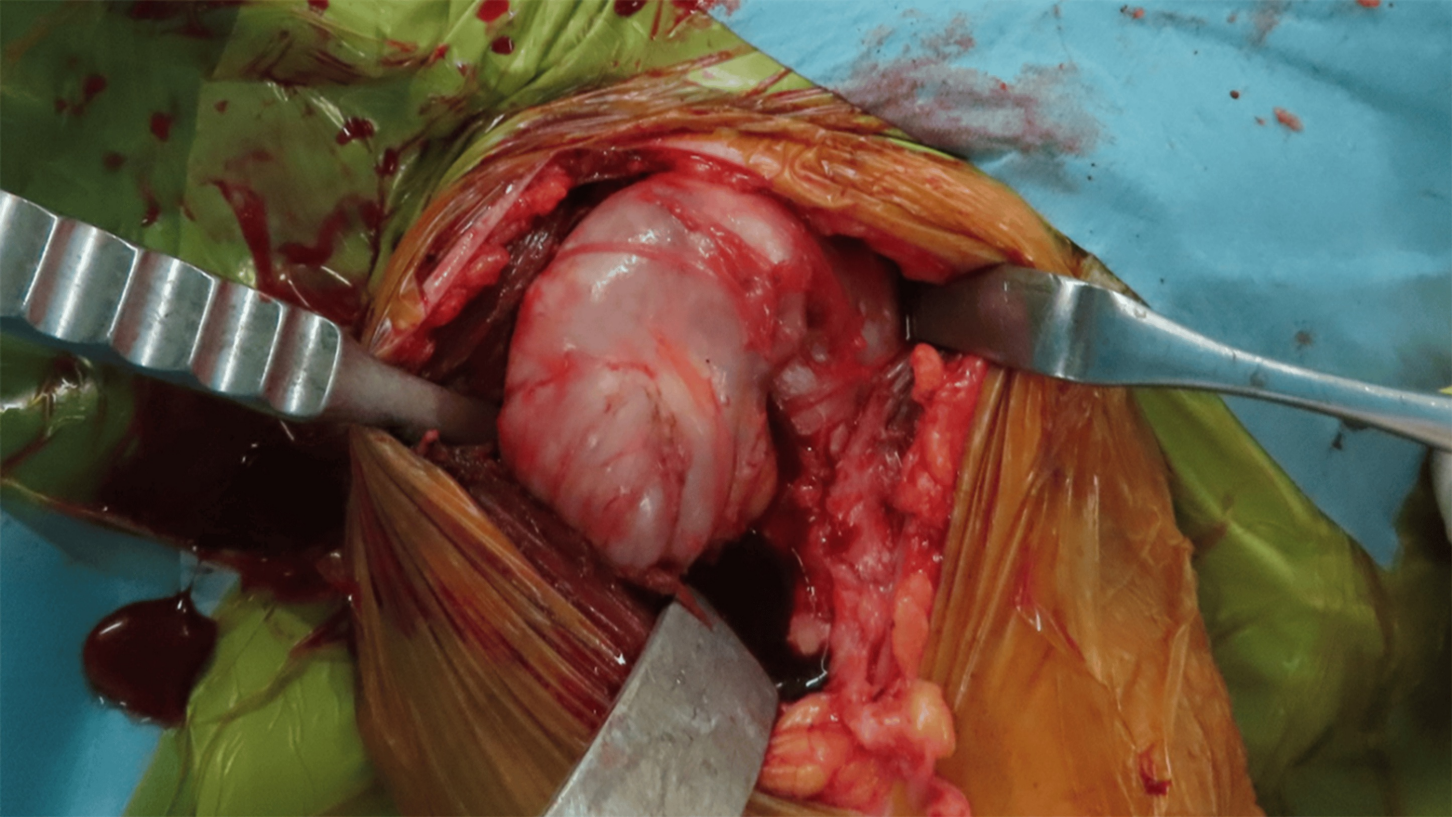
Intraoperative view The subacromial-subdeltoid bursa, including innumerable rice bodies, was observed after splitting between the deltoid and pectoralis major muscles.

**Figure 5:**
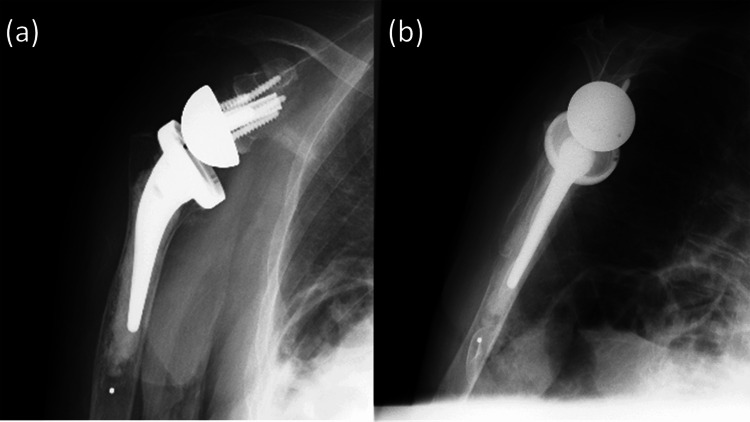
Plain radiographic images on postoperative day 1 (a) Anteroposterior view. (b) Scapular Y view.

After discussion of conservative management, bursectomy, and shoulder arthroplasty, the patient opted for definitive surgical treatment. RSA was performed under general anesthesia using a deltopectoral approach. Upon exposure, a markedly enlarged bursa enveloping the shoulder joint was identified (Figure 4). Incision of the hypertrophic synovial tissue released numerous small, pea-sized loose bodies, all of which were meticulously removed. Communication between the bursa and the joint was confirmed through the full-thickness rotator cuff tear. The humeral head was resected, and the glenoid was prepared for implantation. A reverse shoulder prosthesis with increased bony offset (Aequalis Ascend Flex Reverse; Stryker, Kalamazoo, MI) was implanted using standard techniques (Figure 5). A 7-mm cylindrical autograft harvested from the resected humeral head was used, and a glenoid baseplate with a 25-mm extended central post was selected to optimize host bone contact. Given concerns regarding bone quality related to RA, a cemented humeral stem was chosen to ensure primary fixation. In this case, the supraspinatus tendon was irreparable, and the subscapularis tendon was severely degenerated with poor tissue quality. Therefore, rotator cuff repair was not attempted. In reverse shoulder arthroplasty, joint stability is primarily provided by the deltoid muscle and prosthetic design rather than by the rotator cuff, and non-repair of the subscapularis has been reported to be acceptable in selected cases.

**Figure 6:**
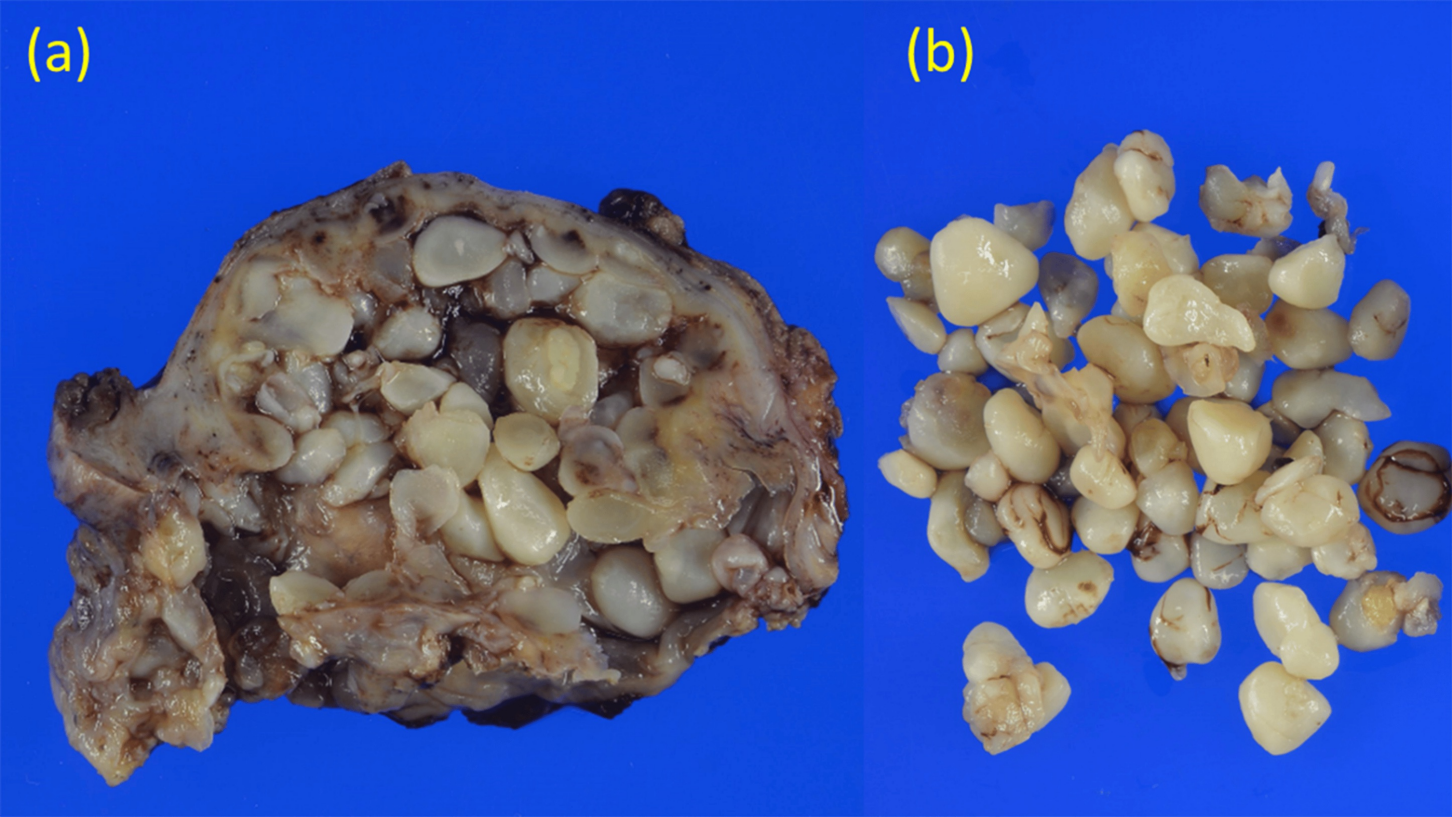
Histopathological examination (a) Gross specimen of subacromial-subdeltoid bursa, including innumerable rice bodies. (b) Gross specimen of extruded-out rice bodies from the bursa.

**Figure 7:**
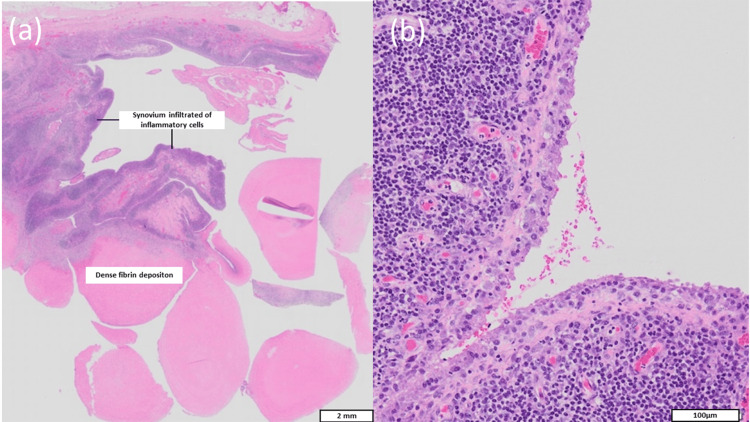
Microscopic histology of rice bodies (a) Rice bodies comprising dense fibrin deposition with synovium infiltrated with inflammatory cells. (b) There is no sign of caseous necrosis.

Histopathological analysis of the bursal tissue demonstrated synovial hyperplasia with chronic inflammatory changes, while examination of the loose bodies revealed fibrinoid material with inflammatory cell infiltration and absence of cartilage or chondrocytes (Figure 6 and Figure 7). Ziehl-Neelsen staining was negative for acid-fast bacilli, supporting the diagnosis of rheumatoid bursitis.

**Figure 8:**
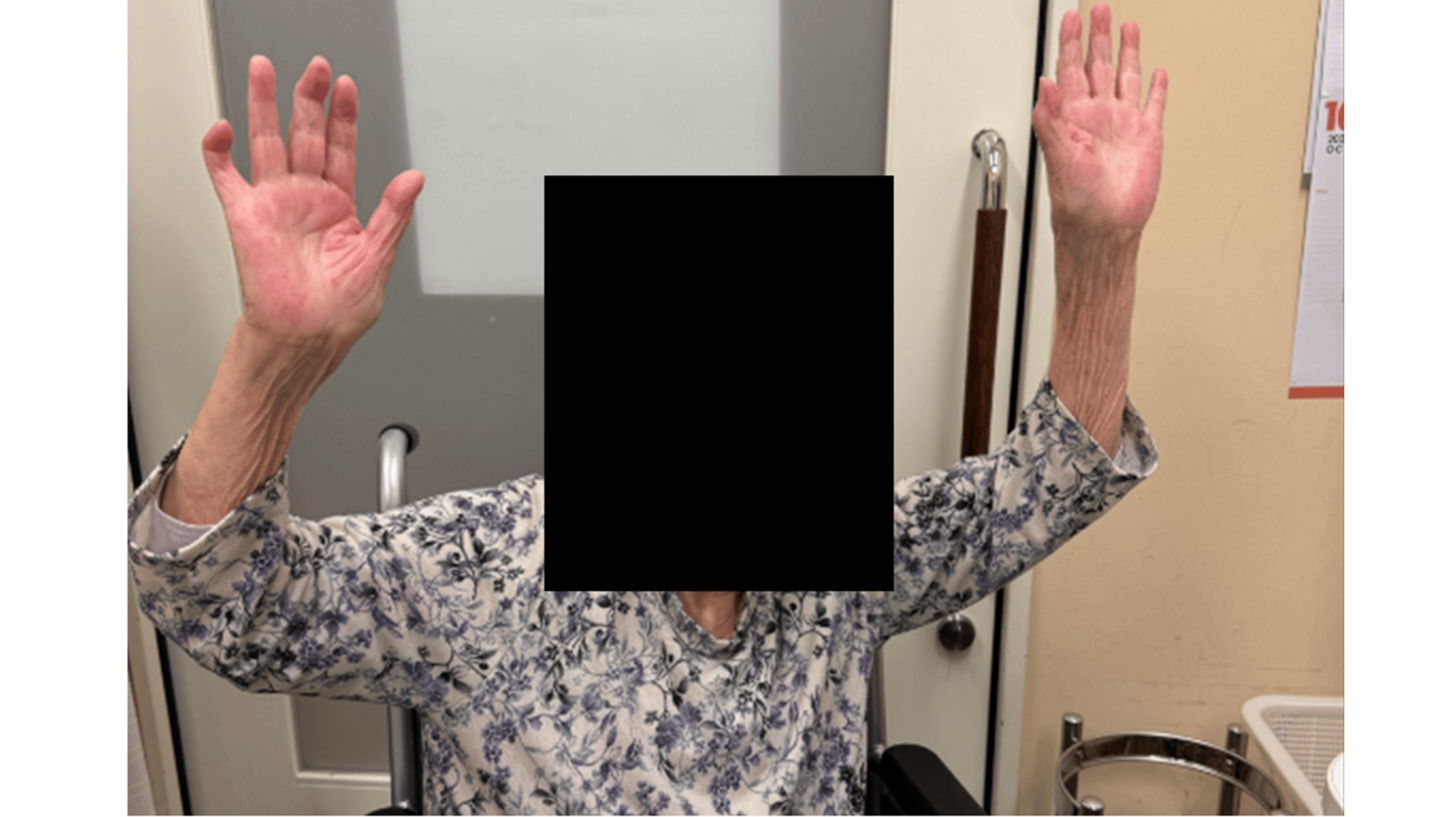
One-year postoperative follow-up of active elevation

Passive range-of-motion exercises were initiated on postoperative day 1, followed by assisted active motion. Wound healing was uneventful. At one-year follow-up, the patient exhibited no recurrence of shoulder swelling and reported painless shoulder motion. RA was well controlled with baricitinib, tacrolimus, and prednisolone, with a C-reactive protein level of 0.62 mg/L. The Constant score improved from 16 preoperatively to 60 points postoperatively (Figure 8).

## Discussion

Rice body formation within joints or bursae is an uncommon condition that was originally described by Reise in association with tuberculous arthritis [12]. Although its precise pathogenesis remains uncertain, rice body formation is generally regarded as an atypical response to persistent synovial inflammation. The majority of reported cases are associated with RA; however, rice bodies have also been observed in nontuberculous mycobacterial infection, seronegative arthritis, juvenile idiopathic arthritis, adult-onset Still’s disease, nonspecific inflammatory arthritis, and osteoarthritis [1,2]. Chronic inflammatory or infectious arthritis and bursitis appear to be common underlying factors [1]. MRI is considered the most useful imaging modality for detecting rice bodies, which typically appear as well-circumscribed nodules with intermediate signal intensity on T1-weighted images and relatively low signal intensity on T2-weighted images. MRI also assists in differentiating rice bodies from pigmented villonodular synovitis and synovial osteochondromatosis, which represent the main differential diagnoses [13]. Histopathological evaluation is essential for the definitive diagnosis of rice body formation, particularly to exclude infectious etiologies such as tuberculosis. Preoperative clinical, laboratory, and imaging findings, however, play a critical role in guiding the initial treatment strategy. In cases where infection is strongly suspected, a staged surgical approach or preoperative biopsy should be considered. Conversely, when clinical, laboratory, and imaging findings do not indicate active infection, single-stage arthroplasty may be a reasonable option. In the present case, histology demonstrated fibrin deposition with inflammatory infiltrates and no evidence of caseous necrosis, while Ziehl-Neelsen staining was negative.

The coexistence of a large Hill-Sachs lesion with RA-related rice body formation is exceedingly rare. In this patient, the Hill-Sachs defect was sufficiently extensive to be classified as off-track and was accompanied by an irreparable rotator cuff tear, both of which significantly compromised shoulder stability. Although rotator cuff tears frequently accompany rice body formation in the shoulder, previous reports have largely described treatment limited to bursectomy without addressing cuff pathology [2,6]. While recurrence of rice bodies appears uncommon following such procedures, persistent functional impairment from untreated rotator cuff tears and the risk of recurrent instability remain concerns. RSA is a semi-constrained arthroplasty that provides stability independent of rotator cuff function. However, patients with RA are known to have an increased risk of complications following RSA due to immunosuppression and reduced bone quality [14]. Lévigne et al. reported favorable outcomes in a multicenter retrospective study of 65 primary RSAs performed in patients with RA, demonstrating a 96% revision-free survival rate at seven years, irrespective of glenoid bone grafting [15]. These findings support the safety and efficacy of RSA in this patient population.

In our case, no evidence of glenoid baseplate loosening, graft resorption, nonunion, infection, or recurrence of rice bodies was observed at one year postoperatively. Adequate control of RA with biological therapy likely contributed to the favorable outcome. RSA effectively addressed all three major pathological factors: rice body formation due to RA, rotator cuff insufficiency, and instability related to humeral bone loss.

## Conclusions

This report describes a rare case of rheumatoid shoulder arthritis with extensive rice body formation, an irreparable rotator cuff tear, and a large Hill-Sachs lesion. Although rice bodies in the shoulder are uncommon and typically confined to the subacromial-subdeltoid bursa, their association with Hill-Sachs lesions has not been previously reported. Reverse shoulder arthroplasty successfully addressed RA-related rice bodies, rotator cuff dysfunction, and shoulder instability due to humeral bone loss, with no recurrence observed at one-year follow-up. Based on this single case, reverse shoulder arthroplasty may be considered as a potential treatment option in carefully selected patients with RA complicated by rice body formation and irreparable rotator cuff tears.

## References

[1] Jeong YM, Cho HY, Lee SW, Hwang YM, Kim YK (2013). Candida septic arthritis with rice body formation: a case report and review of literature. Korean J Radiol.

[2] Qi W, Ren Y, Wang H, Wan Y, Pan H, Yao J (2023). Candida parapsilosis-caused arthritis with rice body formation: a case presentation and literature review. Infect Drug Resist.

[3] Iyengar K, Manickavasagar T, Nadkarni J, Mansour P, Loh W (2011). Bilateral recurrent wrist flexor tenosynovitis and rice body formation in a patient with sero-negative rheumatoid arthritis: a case report and review of literature. Int J Surg Case Rep.

[4] Tian Y, Zhou HB, Yi K, Wang KJ (2022). Idiopathic tenosynovitis of the wrist with multiple rice bodies: a case report and review of literature. World J Clin Cases.

[5] Fujieda Y, Ninagawa K, Matsui Y, Kono M, Kamishima T, Iwasaki N, Atsumi T (2020). Non-tuberculosis Mycobacterium tenosynovitis with rice bodies in a patient with systemic lupus erythematosus. Intern Med.

[6] Rush C, Jochl O, Lowenstein N, Mazzocca J, Matzkin E (2024). Bilateral subacromial-subdeltoid rice bodies in the shoulder: a surgical case report. Case Rep Orthop.

[7] Skelly DL, Konieczko EM, Ulrich J (2023). Rice bodies in a shoulder bursa: a cadaveric and histologic case report. J Man Manip Ther.

[8] Mishra BN, Poudel RR, Jha A, Mainali N, Bhattarai M (2019). Rheumatoid subacromial-subdeltoid bursitis with rice bodies: a case report. J Clin Orthop Trauma.

[9] Joshi PS (2018). Severe sub-acromial bursitis with rice bodies in a patient with rheumatoid arthritis: a case report and review of literature. Malays Orthop J.

[10] Itoi E (2017). 'On-track' and 'off-track' shoulder lesions. EFORT Open Rev.

[11] Hatta T, Yamamoto N, Shinagawa K, Kawakami J, Itoi E (2019). Surgical decision making based on the on-track/off-track concept for anterior shoulder instability: a case-control study. JSES Open Access.

[12] Reise H (1895). The rice corks in tuberculous synoval sacs. Dtsch Z Chir.

[13] Bayoud W, Rizkallah M, Georges S, Younan T, Haykal G (2017). A large rice body-containing cyst mimicking infection following total hip arthroplasty: a case report. Case Rep Orthop.

[14] Cho CH, Kim DH, Song KS (2017). Reverse shoulder arthroplasty in patients with rheumatoid arthritis: a systematic review. Clin Orthop Surg.

[15] Lévigne C, Chelli M, Johnston TR, Trojani MC, Molé D, Walch G, Boileau P (2021). Reverse shoulder arthroplasty in rheumatoid arthritis: survival and outcomes. J Shoulder Elbow Surg.

